# The Higher and Better Education of the Dental Student*Chairman’s address, read before the Section on Stomatology at the Seventieth Annual Session of the American Medical Association, Atlantic City, N. J., June, 1919. Reprinted from *Journal A. M. A.*, September 13, 1919.

**Published:** 1919-10

**Authors:** Eugene S. Talbot

**Affiliations:** Chicago


### THE HIGHER AND BETTER EDUCATION OF THE DENTAL STUDENT *

EUGENE S. TALBOT, M.D., CHICAGO

The object of the formation of this section was to assist in establishing a closer relationship between the mother profession of medicine and the younger specialty of dentistry, and eventually to place the specialty on a medical basis. How much has been accomplished in this direction since its formation, history has shown. Certainly the interest displayed by the ablest men in the medical profession by their presence, in reading and discussing papers, demonstrates that they have done more than their share in placing this section on an equal footing with the other sections in this great association.

In my early practice of dentistry I soon realized that if I was to become a successful practitioner, I must know more of pathology than I had obtained in the dental school. To obtain that knowledge I must attain a medical education; and although it was a hardship for a man with a family to abandon practice for two years, I have been more than compensated by the broader knowledge and fellowship which I have acquired. Every man starting out in life should have high ideals, and those ideals should be so broad and advanced that it requires a lifetime to accomplish the end.

The ideals firmly fixed in the minds of those men who organized this section were that dentistry, being a part of the healing art, should and must be a part of the medical profession, and that a thorough medical education was just as necessary for treating diseases in the mouth as diseases in any other organ of the body. The dental

* Chairman's address, read before the Section on Stomatology at the Seventieth Annual Session of the American Medical Association, Atlantic City, N. J., June, 1919. Reprinted from *Journal A. M. A.*, September 13, 1919.

schools have begun to realize this fact, for since this section was established, the college course has been extended from a two-year to a four-year course, the entire length of a full medical education.

It is rarely the lot of man to live to see his ideals developed to their highest attainments. Nearly all of the organizers of this section have passed on. With one exception, however, they all lived to see great advances along the lines of their ideals.

The stomatologist with his medical education is on an equal footing with the men practicing in every other department of the healing art, and is, therefore, freely consulted in the most difficult and obscure etiologic symptoms of disease in which the mouth, jaws and teeth may possibly be a factor.

As time goes on and the etiology of disease becomes better understood, the relationship of the general practitioner and the stomatologist must become more intimate. It stands to reason, then, that the man who treats the mouth, jaws and teeth must be a medical specialist, equal to the best medical specialist in other branches of the healing art.

It may be of interest to the members present to know that in the thirty-nine years of its existence, this section has not received a single paper on the subject of the mechanics of dentistry. All papers presented and read before the section have been on the subject of pathology or on some subject of interest to both physicians and stomatologists. By adopting this plan, physicians have been as much interested in reading the papers of this as of other sections.

On the other hand, many dentists subscribe for *The Journal* or obtain access to it through physicians in their vicinity. Through this section, therefore, there has developed a closer relationship between medicine and dentistry. The future of this section has a prosperous outlook. Focal infections arising from diseased teeth, and also the associations as a result of the war, have drawn the stomatologist and the medical practitioner into closer relationship. We have arrived at the period when one can not possibly get on without the other. Physicians are seeking specialists who are qualified to assist them in a proper diagnosis of disease, and naturally associate with those who are medically educated. In the future, therefore, the man who wishes to be a success in his specialty must have a medical training.

The future of the section is assured. Dr. Black comes to us as secretary in the fulness of youth and experience. I am sure that he will direct the future welfare of the section to a higher and broader usefulness.

### WOUNDS OF THE FACE AND JAWS

Modern methods of war and its destruction have developed a line of surgery of the face and jaws that could not have been produced in any other way. Injuries due to explosive shells have caused more extensive wounds than can be produced in any other way. These include fractures, destruction of bone structure, and lacerations of the associated soft parts.

We have with us at this meeting stomatologists who have been most active in the different branches of this service and who will demonstrate the latest methods of teaching of the treatment of wounds.

### FOCAL INFECTION

It has been shown and accurately demonstrated that 95 per cent. of all roots of teeth are imperfectly filled. In other words, owing to irritations in the process of treating and filling the roots of teeth or to the decomposition of tissue after filling, pathologic changes occur about the roots, as shown by the roentgen ray and microscope. These pathologic changes have been known to the profession for the past eight or ten years, and yet the same methods of treatment are applied to-day as formerly. How long would the medical profession continue to perform an operation on any other tissue of the body, knowing that the technic was so faulty? My researches on animals during the past twelve years, show that the least irritation produced in the treatment of the roots of the teeth will cause absorption of the alveolar process and roots, with irritation and inflammation of the peridental membrane.

Abnormal collateral arterial development in the roots of the teeth and the abnormal development of the granular layer of Tomes—researches of which have not yet been published—demonstrate the almost complete hopelessness of permanent success, no matter how well the root may have been filled.

The number of focal infections and disturbances from faulty root fillings, local and systemic, is appalling. The average dentist, not being grounded in pathology, does not and can not appreciate the seriousness of his faulty treatment. A different method of root filling must be immediately instituted or devitalized teeth must be extracted. A rule which I have adopted is that a patient's health is worth more than all the natural teeth.

### FAULTY METHODS OF PRACTICE

From time to time, in the past thirty years, I have repeatedly called the attention of the profession to the fact that modern dentistry is producing more disease than any other one cause. The profession is beginning to realize the truth of this statement. Since the first dental college was established, there has gradually developed a method of practice of mechanics regardless of pathologic results. The method of practice, at best, has benefited the individual patient only for the time being. On the other hand, after operations are performed, disease has followed in almost every instance.

Our present method of treatment has not benefited the race as a whole. Decay of the teeth and deformities of the jaws have increased from generation to generation until at the present time hardly a child under 12 years of age can be found free from tooth decay and deformities of the teeth and the jaws. Since our present methods of procedure are faulty, we must resort to radical changes in treatment and devote our entire attention to preventive measures.

### MEDICAL TEACHING VERSUS DENTAL TEACHING

The wonderful progress made in medical teaching in the last fifteen years should be an incentive for the better education of the dentist. In 1904 there were about 156 medical schools in this country. Through the influence of the Council on Medical Education the standard has been raised and the number of schools, by combining one with another or by dropping some out altogether, has been reduced in the last fifteen years to eighty-five. From 80 to 95 per cent. of these schools now require for admission two years of work in a college of arts and sciences, as compared with only 2.5 per cent. of all colleges holding that requirement in 1904. In some of the schools there has been a continuous session of the four quarters plan, whereby the student is allowed to select any three quarters of the year that best suit his convenience to complete his yearly term.

The chairman, Dr. John M. Dodson, in his report asked: "Is there a logical reason why the student should not be allowed to utilize the summer months in full-time medical work in residence and thus complete the present requirement of four sessions in three calendar years, his year of interneship in a fourth year, and so enter into active practice a year earlier than has been possible under previous regulations?"

Under the four quarters scheme, seven weeks may be provided for vacations so that attendance on the four quarters is no hardship for the student in good health who desires to avail himself of the privilege.

Dr. N. P. Colwell, in stating the further needs in medical education, said that an improvement still greatly needed in medical schools concerns the methods of clinical instruction of medical students. Students should be enabled to complete most of their didactic courses in the third year, so that most, if not all, of the time of the fourth year may be devoted to clinical courses for groups of about six students each in the hospital wards.

These changes, so necessary in medical schools, are just as necessary in the dental schools. I have advocated these changes in dental teaching for a number of years. Why should a student spend four years in college when he can save a year far easier than the medical student, since only half of his curriculum is given up to didactic teaching and the other half to the mechanics of dentistry? The mechanics of dentistry can very easily be taught in the summer months, and the fundamental principles of medicine and dentistry may be left for the winter months. The curriculum can be so arranged that the student can have from five to seven weeks' vacation, which is from three to five weeks longer than the vacation of the average professional man.

I have held for many years that the dental school could not make a professional man out of the student by the present method of teaching, even if the course should be extended to six or eight years. There are two grades of dental colleges in the United States: those that are an actual part of universities or colleges, and those that are not. Those that are a part of universities have nothing to do with the matriculation of students. Those that are not have everything to do with the entrance requirements of students. There is an unwritten law among the dental schools that all students entering must be either graduates of a high school or they must successfully pass an examination covering the average high school requirements. The result is that a large percentage of the students entering the dental schools other than those connected with universities or colleges have not an education equal to that of our common schools. It is possible that the deans can explain how these students enter.

Having entered, this uneducated student immediately begins to ascertain what is necessary for graduation. He soon learns that the mechanics of dentistry is the essential thing, since he can not graduate until he can successfully perform certain operations on the teeth and make artificial dentures. Most of his thought and time is spent along these lines. Courses are pursued in the departments of anatomy, physiology, pathology, bacteriology, chemistry, etc., but are not considered of so much importance as it is the mechanics of dentistry that counts toward graduation and practice.

The student begins his mechanical training almost as soon as he enters school. The result is that the ignorant young man, following the line of mechanics, on graduation enters practice as a mechanic rather than as a professional man. As I have already stated, by this method of teaching, a six-year or eight-year course would not improve the mentality or standing of the individual in the community.

Again, students entering the dental school from the farm, shop and factory are unmindful of what is required of them when they enter practice. They pay their fee and expect to be qualified to serve the public successfully when they enter practice. They soon learn, however, that a broader knowledge of the essentials of medicine is necessary to cope successfully with the conditions presented by their patients than they had received in their dental training.

The remedy is easy. Make a professional man out of the dentist, as well as a mechanic. Require the student to take one or two years' work in the arts and sciences. This will relieve the dean of the dental school of all responsibility of the preliminary requirements. The first two years of work in the dental school should be given up to didactic teaching. The student should not be permitted to operate on patients or perform mechanical laboratory work until the third year. All or nearly all of the last two years may be given up to the practical side of the specialty. The preliminary training in the laboratory of the academic and premedical courses will give the student all the finger training that is necessary to prepare him for his dental activities.

### BOOK READERS

There are about 45,000 dentists in the United States. Out of that number not over 500 buy books relating to their specialty, or read them. The reason for this has already been explained. Dentistry is a part of the healing art. There is no reason why the dentist should not be as well educated as the physician and hold an equal position in the community. It is high time that the profession itself awoke to the situation and demanded better qualified graduates from our dental schools.

### UNIVERSITIY SCHOOLS

This section has always used its influence on the side of the university schools. Your secretary has made it a point to attend the annual meetings of the Dental Faculties Association of American Universities when possible, and has always been received with much cordiality. He has read and discussed papers relating to the higher and better education of the dentist. I would suggest that the executive committee of this section be made a standing committte to attend the annual meetings when possible and use its influence on behalf of this section to suggest improvements in special teaching and to encourage the teachers in their efforts to place their schools on an equal footing with other departments of the healing art.

